# Time-Dependent Field Effect in Three-Dimensional Lead-Halide Perovskite Semiconductor Thin Films

**DOI:** 10.1021/acsaem.1c01558

**Published:** 2021-09-29

**Authors:** Anil Reddy Pininti, James M. Ball, Munirah D. Albaqami, Annamaria Petrozza, Mario Caironi

**Affiliations:** † Center for Nano Science and Technology @PoliMi, Istituto Italiano di Tecnologia, via G. Pascoli 70/3, Milano 20133, Italy; ‡ Physics Department, Politecnico di Milano, Piazza L. da Vinci, 32, Milano 20133, Italy; § Chemistry Department, College of Science, King Saud University, Riyadh 11451, Saudi Arabia

**Keywords:** metal-halide perovskites, charge transport, carrier mobility, field-effect transistors, solution-processed semiconductors

## Abstract

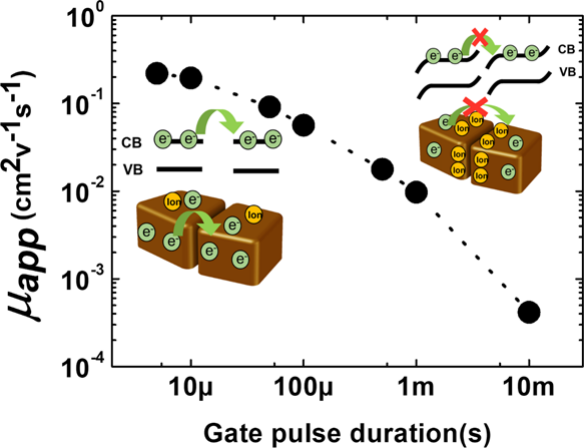

Charge transport in three-dimensional metal-halide perovskite semiconductors is due to a complex combination of ionic and electronic contributions, and its study is particularly relevant in light of their successful applications in photovoltaics as well as other opto- and microelectronic applications. Interestingly, the observation of field effect at room temperature in transistors based on solution-processed, polycrystalline, three-dimensional perovskite thin films has been elusive. In this work, we study the time-dependent electrical characteristics of field-effect transistors based on the model methylammonium lead iodide semiconductor and observe the drastic variations in output current, and therefore of apparent charge carrier mobility, as a function of the applied gate pulse duration. We infer this behavior to the accumulation of ions at the grain boundaries, which hamper the transport of carriers across the FET channel. This study reveals the dynamic nature of the field effect in solution-processed metal-halide perovskites and offers an investigation methodology useful to characterize charge carrier transport in such emerging semiconductors.

## Introduction

Three-dimensional metal-halide perovskite semiconductors have shown tremendous performance in optoelectronic devices, with solar cells achieving laboratory power conversion efficiencies above 25%.^1,2^ Owing to their outstanding properties, such as high charge carrier mobility,^3^ long diffusion lengths,^4^ ambipolar nature,^5,6^ and solution processability, 3D perovskite semiconductors have raised renewed interest also for adoption in field-effect transistors (FETs) after the pioneering work by Kagan et al.^7^ in 1999 on two-dimensional perovskite FETs. While 3D perovskite FETs could be investigated as a possible new powerful candidate for low-cost, large-area, and flexible electronics, it is certainly a powerful platform to deepen the understanding of charge transport in semiconducting thin films.^8,9^ Field-effect measurements can contribute to the understanding of, for example, structure–transport property relationships,^10^ electron–phonon coupling,^11^ and the interplay of electronic and ionic transport,^12^ typically related to a hysteretic behavior.^13,14^ All these aspects also play a relevant role in optoelectronic devices such as photovoltaic cells and light-emitting diodes.^15^

Such expectations have been somehow broken by a series of reports on polycrystalline 3D perovskite films where the room-temperature field-effect behavior could not be observed.^16−18^ A clear field-effect behavior could be instead observed for temperatures lower than 220 K, with performances gradually degrading when approaching room temperature.^16−19^ Such observations were interpreted both as an effect of electron–phonon coupling^16^ and ion migration, hindering accumulation and transport of electronic carriers at room temperature.^16,20,21^ In particular, ionic species migration at room temperature is easily observed owing to the low activation energy of mobile ionic species.^22^

Yu et al. recently reported successful room-temperature operation of 3D perovskite FETs utilizing in situ-grown MAPbX_3_ (methyl ammonium lead trihalide) single crystals (X = Cl, Br, and I).^23^ Very good ambipolar room-temperature FET characteristics, with electron and hole mobilities up to 1.5 and 4.7 cm^2^ V^–1^ s^–1^, were observed. Such evidence strongly suggests that grains and grain boundary defects limit the polycrystalline 3D perovskite FET performance at room temperature. In fact, in solar cell devices, based on polycrystalline films, charges are transported within hundreds of nanometers thick grains interconnecting bottom and top charge extraction layers in a vertical structure. Instead, in typical planar FETs, transport occurs in the perpendicular, lateral direction, with charges encountering several grain boundaries along micron-long channels.^24^

It has to be noted that a few isolated cases report evidence of room-temperature field-effect behavior also in solution-processed polycrystalline 3D perovskites.^25−27^ However, no evidence or conclusive reason for such an observation has been proposed. So far, room-temperature field-effect behavior in polycrystalline 3D perovskite films is an elusive phenomenon, and the contributions of structural defects and mixed electronic and ionic transport are expected to play a relevant role.^24,28,29^

In order to allow the investigation of the electrical characteristics of polycrystalline perovskite FETs in the presence of such combined effects, Labram and co-workers were the first to adopt transient measurements, in particular, for the case of a model MAPbI_3_ system.^18^ In their case, clear electronic transport owing to the field effect could be observed for temperatures as high as 220 K by applying gate voltage pulses lasting a few seconds. A similar strategy was later proposed by Senanayak et al.,^9,30,31^ who investigated solution-processed MAPbI_3_ FETs by adopting 500 μs-long gate pulses. Thanks to this approach, they successfully reported room-temperature n-channel operation, with a claimed electron mobility of 0.5 cm^2^ V^–1^ s^–1^.^9^ The much shorter pulse duration with respect to the study by Labram et al. is a reasonable hypothesis for the explanation of the different results at room temperature. More recently, a recent report by Maddalena et al. consistently reported room-temperature operation of a 3D perovskite light-emitting FET by adopting pulsed mode operation with pulses as short as 100 μs.^32^

Pulsed measurements are therefore a useful tool to investigate 3D perovskite thin films in a field-effect experiment. By applying voltage pulses to the gate electrode, instead of a continuous bias, it is possible, for example, to gain information of concurrent processes characterized by different timescales as for example the accumulation of an electronic channel with respect to ion migration. However, little to no attention has been paid so far to the duration of pulses, and the choice of the timescale appears arbitrary or possibly related to instrumental limitations. This is a critical aspect, also evidenced in a recent review paper on the topic,^33^ as the measured currents and the derived parameters may strongly vary with the adopted pulse duration if concurring effects with different timescales are present, as those characterizing electronic and ionic transport, as well as charge carrier trapping and detrapping events. Hence, a detailed investigation of the time-dependent field-effect behavior in 3D perovskite films is essential to elucidate the validity and limits of pulsed measurements. In this study, we have directly addressed this issue, revealing the dynamics of the electronic channel accumulation and decay with time in field-effect devices based on solution-processed MAPbI_3_ thin films. Our results make it evident that the MAPbI_3_ FET characteristics depend on the gating time and therefore require a detailed temporal analysis.

**Figure 1:**
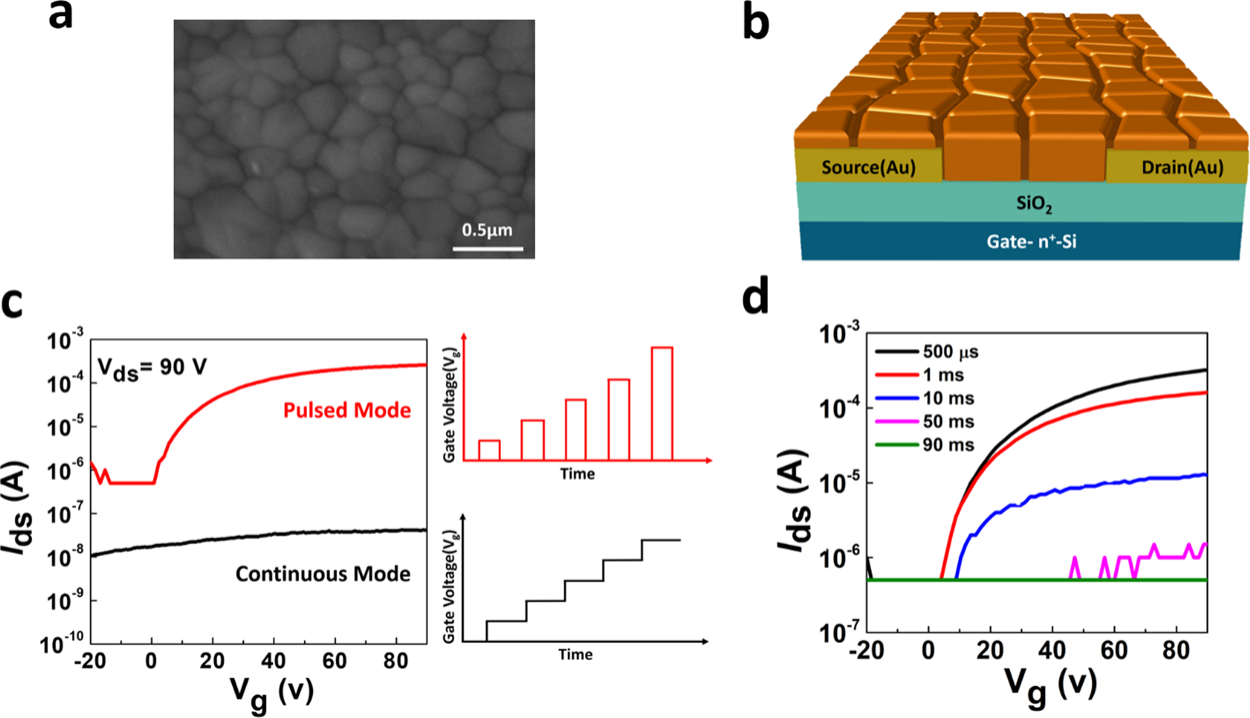
(a) Top-view scanning electron microscopy (SEM) micrograph of an MAPbI_3_ thin film. (b) Schematic representation of the FET adopted in the study, with *L* = 10 μm and *W* = 10 mm. (c) Continuous mode (black; each step of the staircase is held for 0.3 s; each step increment is 0.6 V; and the measurement scan rate is 2 V/s) and pulsed mode (red; pulse width: 500 μs; and repetition period: 100 ms, corresponding to a duty cycle of 0.5%) FET transfer characteristic curves measured at room temperature with *V*_ds_ = 90 V. (d) FET transfer characteristics with the pulse duration increasing from 500 μs to 90 ms.

For our investigation, we selected solution-processed MAPbI_3_ thin films, grown by single-step spin coating of a 0.75 M perovskite precursor solution (detailed in the Experimental Section). The resulting perovskite films are 150 nm thick with a lateral grain size up to about 400 nm (Figure 1a). With such films, we fabricated bottom-gate, bottom-contact FETs, characterized by gold-interdigitated source-drain electrodes, with a channel length (*L*) of 10 μm and a width (*W*) of 10 mm, and by a SiO_2_ dielectric on top of heavily n-doped Si as the gate electrode (Figure 1b).

The electrical characteristics of the fabricated FETs were measured at room temperature in the dark and in a nitrogen environment to avoid any external influence on the device performance. The transfer characteristics were acquired at a fixed drain-source voltage (*V*_ds_) and with a varying gate-source voltage (*V*_gs_) according to two schemes to directly compare the results (Figure 1c): (i) a continuous mode scheme, where *V*_gs_ is applied according to a staircase and therefore a vertical field is always present along the measurement and (ii) a pulsed mode scheme, where *V*_gs_ is applied in consecutive pulses in between which the gate voltage is turned off. In this case, we adopted 500 μs long pulses, applied every 100 ms, as done in previous studies adopting the pulsed mode.^9^Figure 1c shows the comparison of the drain currents measured with the two modes. In the continuous mode, no field effect can be observed, and the measured current shows eventual variations that are only time-dependent and completely unrelated to the varying *V*_gs_. This is consistent with several previous reports on polycrystalline MAPbI_3_ at room temperature.^16−18^ Instead, in the pulsed mode, a clear n-type field-effect response can be recorded, indicating the formation of an accumulated electron channel that modulates the conductivity. This evidence qualitatively confirms the result obtained by Senanayak et al.^9^ with the same pulse duration. In our case, from the transfer characteristics, adopting a gradual channel approximation, an electron apparent mobility (μ_app_) of 0.018 cm^2^ V^–1^ s^–1^ can be derived.

The obvious question at this point is which transfer curve would be recorded for different pulse durations and how representative is of the electronic properties of the MAPbI_3_ film a mobility value derived by setting a fixed 500 μs long pulse. With the increasing pulse duration from 500 μs to 90 ms, the channel current keeps decreasing until no field effect is observable any more at 90 ms (Figure 1d). This shows evidence of the strong time dependence of transfer curves measured in the pulse mode and a first indication on the timescale of superposition of different effects; consistently with measurements in the continuous mode, above 90 ms, no electronic modulation of the channel current takes place anymore.

The absence of measurable field effect at room temperature, above 90 ms and in the continuous mode, can be ascribed to ion migration. Halides and methylammonium defects are the most probable migrating ions as their activation energy is calculated to be as low as ∼0.1 eV for the iodide vacancies, 0.5 eV for MA^+^ vacancies, and 0.8 eV for Pb^2+^ vacancies.^29,34,35^ Such species can either accumulate in response to the applied field, thus screening the gating, or form energetic barriers for intergrain transport of electronic charges when accumulating at grain boundaries.^21,36,37^ When a short enough pulse is applied, the modulation of the electrical conductivity of the perovskite film is measurable since the time scale of the electronic channel accumulation is much faster than the ion migration one. The observation of the electronic field effect is therefore possible due to distinct dynamics of ions and electrons. However, with the measuring scheme applied so far, no quantitative knowledge is available for such dynamics.

**Figure 2:**
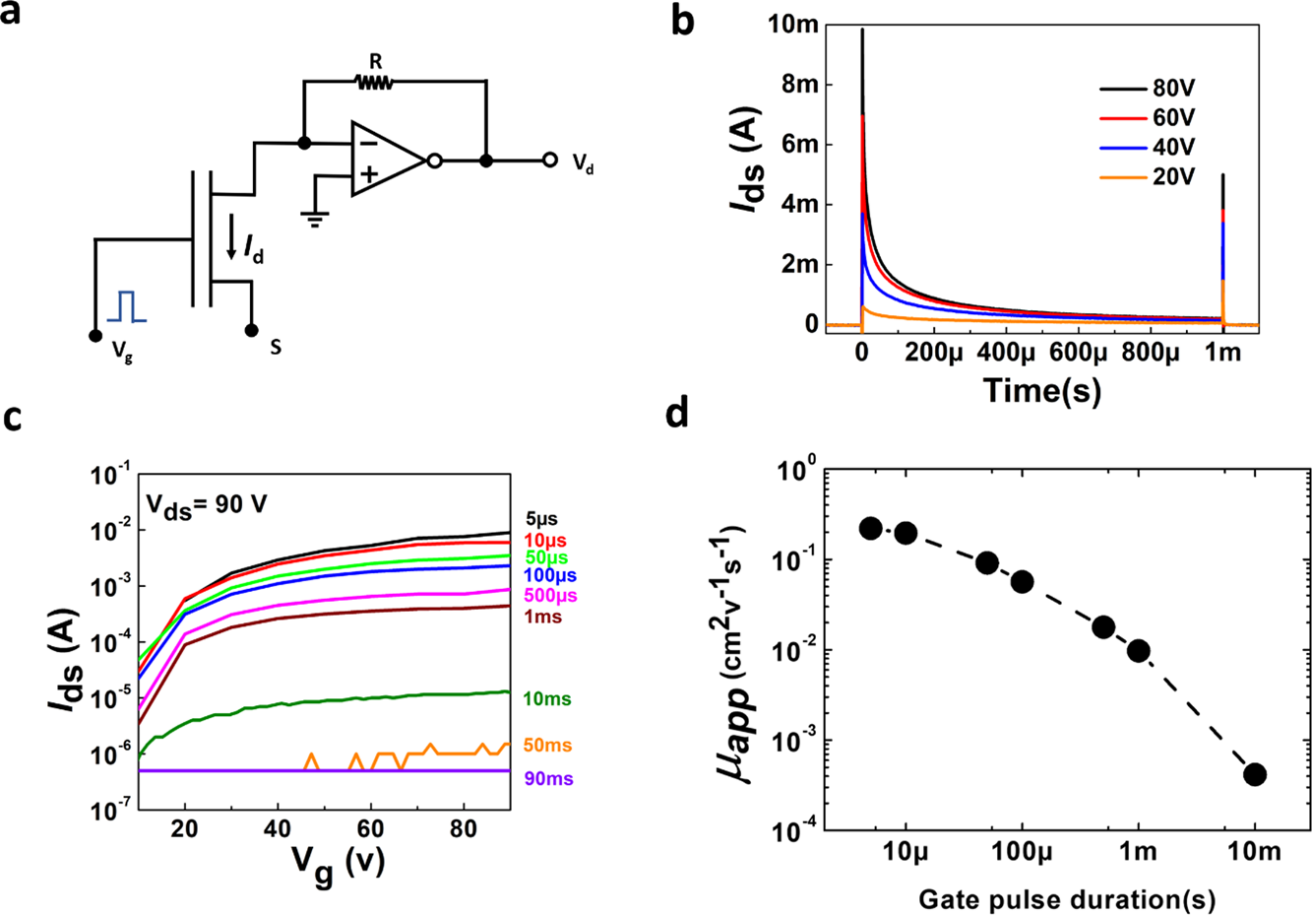
(a) Circuit configuration for transient measurements: a voltage pulse is applied to the gate and the transient channel current is measured, thanks to the transimpedance amplifier; a *V*_ds_ voltage of 90 V is applied by setting *V*_s_ to −90 V. (b) Channel current (*I*_ds_) transient measurements for 1 ms long pulses with varying gate voltage amplitudes. The negative and positive spikes at the start and end of the pulse are due to capacitive coupling. (c) Equivalent transfer characteristic curves obtained by replotting the data of panel (b) as a function of the gate voltage, at different times, from 5 μs to 1 ms; transfer curves directly measured in the pulsed mode at various pulse widths are also presented from 10 to 90 ms to show the consistency for the two different sets of data. (d) Apparent mobility as a function of pulse duration.

Accessing directly the dynamics at the base of the pulse duration dependence and decoupling the electronic field effect with respect to the competitive mechanisms are therefore essential. To do so, we have conducted a transient measurement by means of the setup schematically shown in Figure 2a, where the transient channel current of the FET (*I*_ds_) is recorded upon the application of a pulsed gate voltage. The time domain response is shown in Figure 2b for different *V*_gs_ values. The signal increases sharply upon application of the gate voltage, reaching 9.5 mA at *V*_gs_ = 80 V. Then the current decays with time, reaching 200 μA after 1 ms. The intensity of the recorded signal clearly depends on *V*_gs_. This is a direct observation of the time-dependent modulation of the channel conductivity through the field effect. Electrons can be accumulated at time scales where ionic effects are not already overwhelming such processes, and this is the reason of the fast, gate-dependent increase of the channel current. Then the following decay derives from the competitive effect suppressing the field effect at room temperature. This is the first evidence of such a dynamic process taking place with gating of 3D perovskite transistors, which gives an immediate explanation of the pulse width-dependent transient curves.

The same transient data raise a warning with respect to extrapolation of mobility data from pulsed mode characterization. From Figure 2b, it is possible to reconstruct equivalent FET transient characteristic curves as a function of gating time. These curves are shown in Figure 2c along with those obtained with pulsed mode for comparison, covering a wide range, from 5 μs to 90 ms. The same trend is observed within the whole-time range, the shorter the gating time, the higher the recorded current. The increase slows down with shorter times, indicating saturation of the electronic current at timescales of few microseconds, where ionic interference cannot take place. Such a saturation value would be the one observable in the continuous mode if a stable electronic channel accumulation was achieved. Time-dependent apparent mobilities (μ_app_) can be extracted from each transfer characteristic curve (Figure 2d). Consistently with the channel current, μ_app_ varies approximately over three orders of magnitude, from 0.22 cm^2^ V^–1^ s^–1^ for 5 μs gating to ∼4 × 10^–4^ cm^2^ V^–1^ s^–1^ for 10 ms. Above 10 ms, no field-effect mobility can be extracted. Previous studies reported apparent mobility for MAPbI_3_ FET devices in the pulsed regime, for either 100 or 500 μs pulse duration.^29,31^ It is evident that such apparent mobility is relevant only if combined with the timescale at which it is extracted.

**Figure 3:**
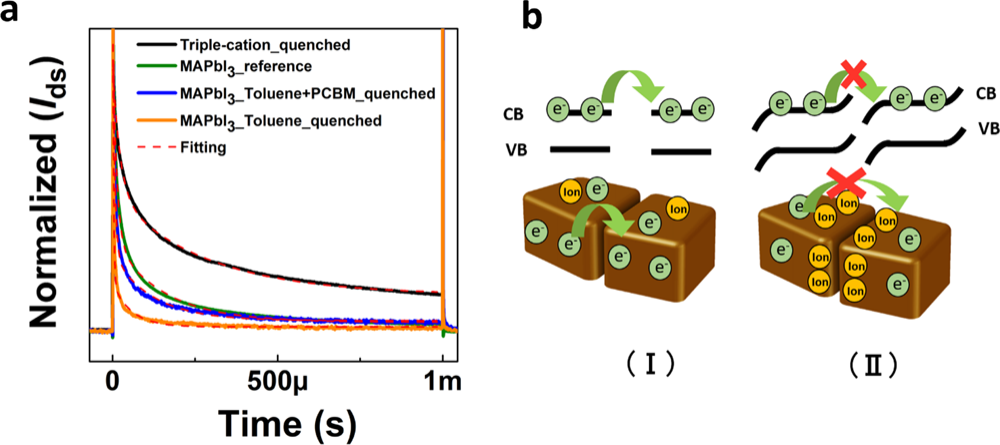
(a) Channel current (*I*_ds_) transient measurements for a 1 ms long pulse. The perovskite thin films have been fabricated according to different methods. (b) Schematic representation of the proposed undergoing mechanism: at shorter times, ions are not yet accumulated at grain boundaries; therefore, electron transfer from one grain to another cannot be affected; at longer times, accumulation of ions at grain boundaries leads to band bending and the formation of energetic barriers for intergrain electron transport, suppressing the observable field effect.

To check the effect of the nature of the perovskite semiconductors on the recorded transient current, we have modified in various ways the active layer in the device. First, we have adopted a solvent quenching methodology (detailed in the Experimental Section), where a 5 mg/mL phenyl-C60-butyric acid methyl ester (PCBM) solution in toluene was casted on top of the perovskite film during spin coating of the perovskite to diffuse PCBM among the grains. It is widely known that incorporation of PCBM within the perovskite system enables passivation of the defects at grain boundaries, which significantly suppresses ion migration.^35,38^ Transient measurements (Figure 3a) performed on the PCBM-passivated MAPbI_3_ FET show a significant difference with respect to the neat MAPbI_3_ case. The presence of PCBM slows down the current decay, an effect consistent with defect passivation, which in turn reduces ion migration.

As an additional test for the dependence of the transient channel current on the level of defectivity, we tested a FET based on a triple-cation perovskite, Cs_*x*_(MA_0.17_FA_0.83_)_1–*x*_Pb(Br_0.17_I_0.83_)_3_, where *x* is 5%. Such a triple-cation perovskite is known to be less prone to defects and ion migration issues.^5^ Also in this case, a slower current decay is consistently observed with respect to the reference MAPbI_3_ FET (Figure 3a), with a dynamic which is also slower than the one for PCBM-passivated MAPbI_3_.

To further monitor the effect of different thin-film properties on the dynamics of the FET, we fabricated a device with the same MAPbI_3_ but adopting a solvent quenching protocol that produces smaller grains and therefore increases the number of grain boundaries. The FET based on the thin film with smaller grains shows a much faster current decay with respect to the reference MAPbI_3_ FET (Figure 3a).

Further studies are necessary to quantitatively analyze the different decays and understand in detail how they depend on the properties of the perovskite thin films. However, our results provide a first clear indication that the characteristic decay times depend both on the level of defectivity and on the grain size. Moreover, the evidence provided also throws some light on how ions respond to applied fields in a FET device. It is evident from transient measurements that at short time scales (i.e., microseconds after the application of a gate bias), an electronic channel can be formed. This is reasonable as ions are not fast enough to respond to the vertical gate field, and an electronic accumulated channel can form. The following decay of the electronic current therefore cannot be simply explained with screening of the gate field by ions accumulating at the interface as the vertical field is screened by the electronic channel first. Our hypothesis is that the decay is due to energetic barriers building up at grain boundaries as an effect of ion accumulation (the detail of two contiguous grains in a polycrystalline 3D MAPbI_3_ perovskite film is sketched in Figure 3b). In FET devices, charges are laterally transported. Electrons flowing along the channel under the applied *V*_ds_ have to overcome several grain boundaries, which is expected to limit and control the overall charge-transport properties. The energetics at such boundaries can be strongly modified by the presence of space charges. In fact, several studies in the literature have reported that ions can migrate and accumulate at such boundaries.^37,39,40^ We speculate that when the FET is switched off (i.e., no gate voltage is applied), the channel is overall very resistive and there is no driving force for ion accumulation at grain boundaries. Instead, when the gate voltage is applied, the grains become much more conductive following electron accumulation, and the lateral field localizes at the grain boundaries, driving the accumulation of ions with time. Ions can thus cause a change in the local electric field at the boundaries, introducing substantial energetic barriers for intergrain electron transport.^37,41,21,42^ When the gate pulse duration is shorter than the characteristic time necessary for ion buildup, the intergrain transfer of electrons is still possible and the field-effect behavior can be observed. From previous literature studies, we note that the positive effect of PCBM can be assigned to its capability of withdrawing iodine.^35,43^ Indeed, in our general picture of Figure 3b, the barriers to extraction of electrons from grains are caused by the negative space charges at boundaries following anion accumulation. Such an effect can be extended to a barrier developing between the semiconductor and the drain contact and limiting electron extraction. While, on the basis of the data reported here, other effects interfering with the electronic field effect cannot be excluded, we note that the observation of FET operation for single-crystal perovskites at room temperature^23^ is consistent with the proposed picture.

## Conclusions

In summary, we have revealed the dynamic nature of the field effect in solution-processed 3D perovskite FETs, and we have proposed the adoption of transient measurements to study such an otherwise elusive phenomenon. Pulsed measurements adopted to circumvent the issue produce FET characteristic curves that depend on the applied gate pulse duration and as such provide a very limited description of undergoing mechanisms and raise a warning with respect to the reliable extraction of physical quantities such as electronic charge mobility. A dynamic investigation of the FET electronic current reveals that in solution-processed perovskite polycrystalline thin films, an electronic current can be readily observed, decaying with characteristic times depending on the level of defectivity and grain size of the semiconducting film. As a consequence, any device parameter, such as the apparent mobility, depends on the time scale adopted. In MAPbI_3_ FET, it varies approximately over three orders of magnitude, from 0.22 cm^2^ V^–1^ s^–1^ for 5 μs gate pulses to ∼4 × 10^–4^ cm^2^ V^–1^ s^–1^ for 10 ms pulses. We suggest ion migration, accumulating at grain boundaries and producing energetic barriers for intergrain electron transport, as the competing mechanism to the field effect. In addition to proposing a useful technique to further investigate the interplay of electronic and ionic charge transport in perovskite semiconductor thin films, this study urges the adoption of standardized methods with clear indications of gating regime and timescale when investigating 3D perovskite FETs. Device parameters extracted with arbitrary choices over pulse widths in the pulsed mode characterization provide very limited information on the thin-film properties that should not be compared with parameters extracted in the continuous mode or with different pulse durations. Such practices will allow the correct evaluation of field-effect properties and the understanding of the interplay of the microstructure, defects, and electronic and ionic transport in the relevant class of perovskite semiconductors.

## Experimental Section

Methylammonium iodide (MAI) and lead acetate trihydrate (≥99.99% trace metal basis) were purchased from Sigma-Aldrich. All materials were used as received without any further purification. Prepatterned source(S)-drain(D) electrodes (ITO/Au =10 nm/40 nm) on Si/SiO_2_ wafers were purchased from Fraunhofer IPMS.

To prepare 0.75 M concentration of a precursor solution, MAI (357.69 mg) and lead acetate trihydrate (PbAc 3H_2_0, 284.5 mg), in a molar ratio of 1:3, were added in 1 mL of *N*,*N*′-dimethylformamide. Later, HPA (6.16 μL) was added to the above solution at a concentration of 2 mol % of MAI. After the dissolution of precursors, the solution was filtered with a 0.45 μm PTFE filter before spin coating.

Au-patterned S-D electrodes (channel length *L* = 10 μm and width = 10 mm) on Si/SiO_2_ substrates were cleaned by sequential sonication with acetone and IPA using an ultrasonic bath for 5 min each and then dried by N_2_ gas flush. Later, substrates were plasma-treated for 10 min. The prepared perovskite precursor solution was spin-coated on Si/SiO_2_ substrates at 4000 rpm for 40 s and annealed at 100 °C (15 min) in a N_2_ glove box atmosphere.

Additionally, the perovskite film MAPbI_3_ and the triple-cation Cs_*x*_(MA_0.17_FA_0.83_)_(1–*x*)_Pb(I_0.83_Br_0.17_)_3_ (*x* = 5%) film from solvent quenching approach were prepared according to procedures largely described in the literature. A volume of 5 mg/mL PCBM in toluene was also used for solvent quenching following a similar approach used for solvent quenching.

## Supporting Information Available

Experimental section, material details, solution preparation and device fabrication, equipment used, material characterization, extraction of mobility, data acquisition points, charging and discharging, MAPbI_3_ FET and organic FET transient responses, reliability tests, SEM cross section, and gate leakage current (PDF)
