# Supplementary material for: Time-Dependent Field Effect in Three-Dimensional Lead-Halide Perovskite Semiconductor Thin Films
Source: ACS Appl Energy Mater. 2021 Sep 29;4(10):10603–9. doi: 10.1021/acsaem.1c01558 (PMC8552216; doi:10.1021/acsaem.1c01558)
Supplement: Supplementary file 1 — ae1c01558_si_001.pdf [file ae1c01558_si_001.pdf]

SUPPORTING INFORMATION

Time-dependent field-effect in three-dimensional lead-halide perovskite semiconductors thin-films

Anil Reddy Pininti[1,2] , James M. Ball[1], Munirah D. Albaqami[3], Annamaria Petrozza [1,3], and Mario Caironi[1] **

1. Center for Nano Science and Technology @PoliMi, Istituto Italiano di Tecnologia, via G. Pascoli 70/3, 20133, Milano, Italy.

2. Physics Department, Politecnico di Milano, Piazza L. da Vinci, 32, 20133 Milano, Italy.

3. Chemistry Department, College of Science, King Saud University, Riyadh 11451, Saudi Arabia

* annamaria.petrozza@iit.it, mario.caironi@iit.it

Section S1. Experimental Section

S1.1 Materials Details

Methylammonium Iodide (MAI) and lead acetate trihydrate (≥99.99% trace metals basis) were purchased from Sigma-Aldrich. All materials were used as received without any further purification. Prepatterned source(S)-drain(D) electrodes (ITO/Au =10nm/40nm) on Si/SiO_2 wafers were purchased from Fraunhofer IPMS.

S1.2 Solution Preparation and device fabrication

To prepare 0.75M concentration of precursor solution, Methylammonium Iodide (MAI) (357.69 mg) and lead acetate trihydrate ratio (PbAc.3H20) (284.5 mg) in the molar ratio of 1:3, were added in 1ml of N,N'–dimethylformamide(DMF). Later, HPA(6.16ul) was added to the above solution at a concentration of 2 mol% of MAI. After the dissolution of precursors, solution was filtered with 0.45 μm PTFE filter before spin-coating.

Au-patterned S-D (channel length L=10um and width= 10mm) on Si/SiO_2 substrates were cleaned by sequential sonication with acetone, IPA using ultrasonic bath for 5minutes each and then dried by N_2 gas flush. Later substrates were plasma treated for 10minutes. Prepared perovskite precursor solution was spin-coated on Si/SiO_2 substrates at 4000rpm for 40 secs and annealed at 100˚C (15 minutes) in N_2 glove box atmosphere.

Additionally, perovskite films $MAPbI_3$ and triple cation $Cs_x(MA_{0.17}FA_{0.83})_{(100-x)}Pb(I_{0.83}Br_{0.17})_3$ (x=5%) films from solvent quenching approach were prepared as per discussed in the literature. [1] [2]

S1.3 Equipment used

All the electrical characterizations were performed using Agilent B1500A semiconductor parameter analyser (SPA) and FLC electronics waveform generator WFG600 pulse generator (PG). All characterizations performed in this paper were carried out in dark and N_2 atmosphere to avoid undesirable effects of ambient atmosphere on devices.

Section S2. Characterization

S2.1 Material Characterization

X-Ray diffraction (XRD) patterns were performed using a BRUKER D8 ADVANCE diffractometer (Bragg-Brentano geometry) with parameters Cu Kα1 (λ = 1.544060 Å) anode, operating at 40 kV and 40 mA. Spectra was captured using 0.05° angular steps with 1s integration.

Scanning Electron Microscope (SEM) images were taken with a Tescan for cross-sections. A Jeol JSM-6010LV at 10 kV acceleration was used to acquire top views.

Atomic Force Microscopy (AFM): The surface topography of the films was measured with an Agilent 5500 Atomic Force Microscope operated in the acoustic mode.

All the electrical characterizations were performed using Agilent B1500A semiconductor parameter analyzer (SPA). For lower pulse width pulsed measurements, FLC electronics (WFG600) waveform was utilized and drain currents were monitored with oscilloscope via impedance amplifier. All characterizations performed in this paper were carried out in dark and N2 atmosphere to avoid undesirable effects of ambient atmosphere on devices.

Section S2.2 Extraction of Mobility

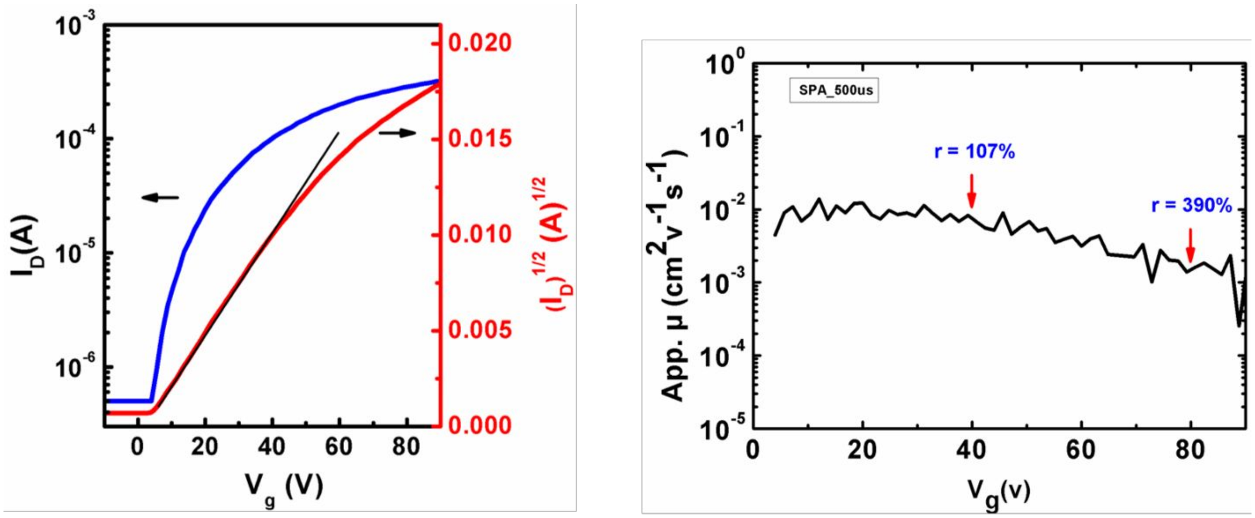

Figure S1. a) Transfer characteristics of SPA (500us) b) Apparent Mobility vs pulsed gate Vg

Fig S1.a illustrates the non-linear behavior of $I_D^{1/2}$ over V_g>40v, consequently mobility also varied with increase of V_g (see fig S1.b). This generates the doubt of extracting mobility. Hyun Ho choi et al. reported an article detailing how to avoid common pitfalls and shown best practices for extracting FET mobilities.[3] Here they have introduced a new parameter, measurement reliability factor(r), which is the ratio of experimentally achieved maximum channel conductivity experimentally achieved to the maximum channel conductivity of an equivalent ideal FET. Transistors performances near to ideal characteristics, show 'r' values around 100%, suggests most unambiguous mobility extraction. In this work till V_g=40v, sqrt Id is linear, and the calculated 'r' value is 107% which hints to reliable mobility extraction. But when V_g> 40v, 'r' value increases up to 300% (at V_g=80v), which hints in erroneous mobility extraction. Hence following Hyun Ho

choi et al. guidelines, in this article, we have extracted all mobilities in the region where r value nearly equal to 100% which is under V_g<40v. Since all the pulse width characteristics follows the same trend, for reference we have shown mobility extraction for only pulse width 500μs (SPA). Mobilities are extracted by equation.

$$\mu = \frac{2L}{WC}\left(\frac{\partial\sqrt{Id}}{\partial V_{gs}}\right)^2$$

Section S2.3. Data Acquisition points

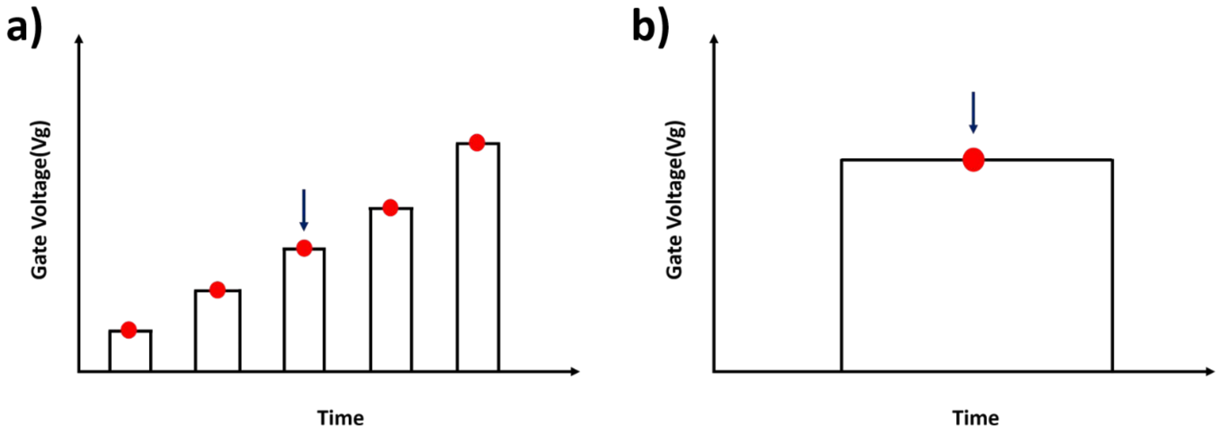

Figure S2. Data points acquired at the center of pulse (red dots) for a) SPA and b) PG.

For all pulsed measurements conducted in this article, current I_D data points are acquired from the center of Gate voltage pulse.

Section S2.4. Charging and discharging

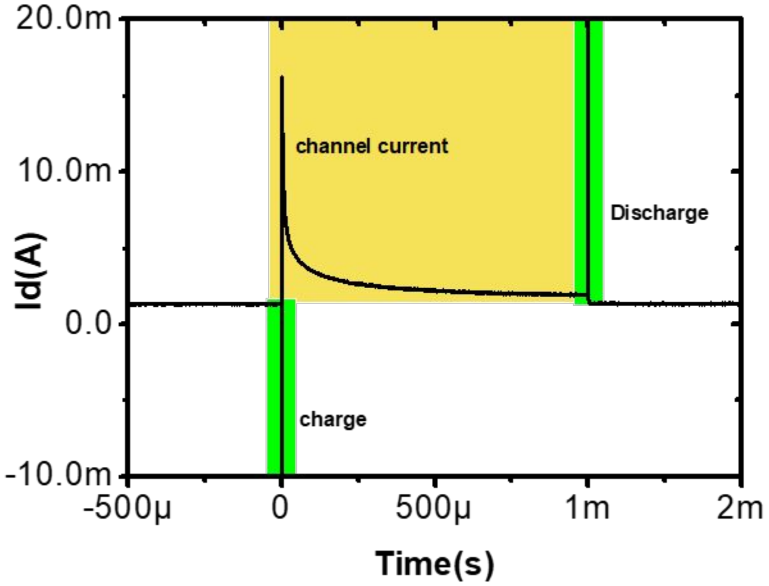

Figure S3. 1ms single gate-pulse (color highlighted region) transient measurement showing charging and discharging peak (green peaks)

From fig S3 it is clearly illustrated the existence of two peaks one just before beginning and other just after the end of the pulse. These peaks are ascribed as the charging and discharging peaks and noted that they are not participating during the active pulse width. Also, data points are acquired from the center of the pulse, hence FET characteristics obtained are not influenced by this charging and discharging peaks

Section S2.5. $MAPbI_3$ FET and organic FET transient response

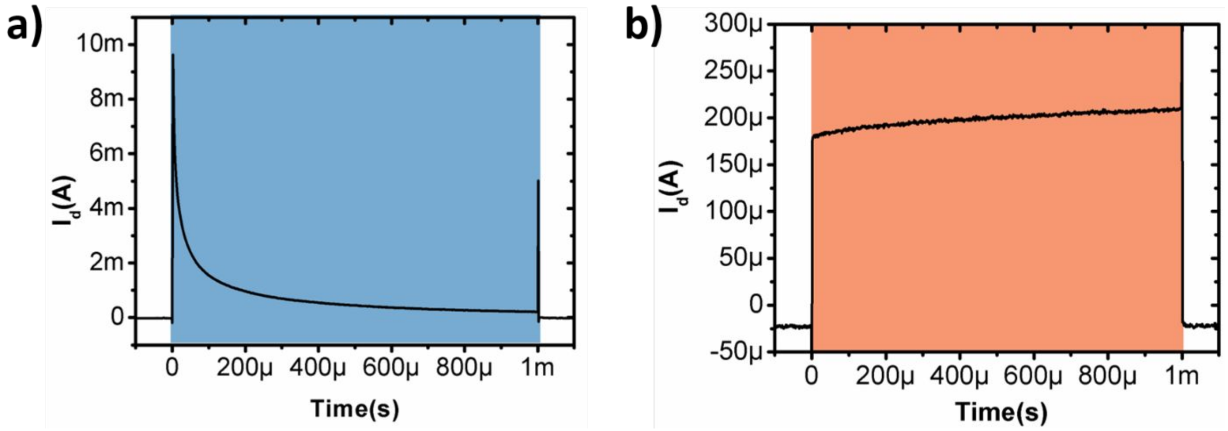

Figure S4. a) perovskite ($MAPbI_3$) FET b) Organic(N2200) FET single pulse transient measurement (1ms) (color highlighted region)

We have conducted transient measurements (pulse width (1ms)) on $MAPbI_3$ (see fig. S4.a) and organic FETs (see fig. S4.a) to demonstrate the variation of their performances. Fig S4 clearly illustrates, decay of drain current with pulse width increment in $MAPbI_3$ FETs, whereas organic FETs showed constant drain current without any decay. This clearly suggest that, ion migration in perovskite influences the FET characteristics.

Section S2.6. Transient response by different perovskite composition and approaches

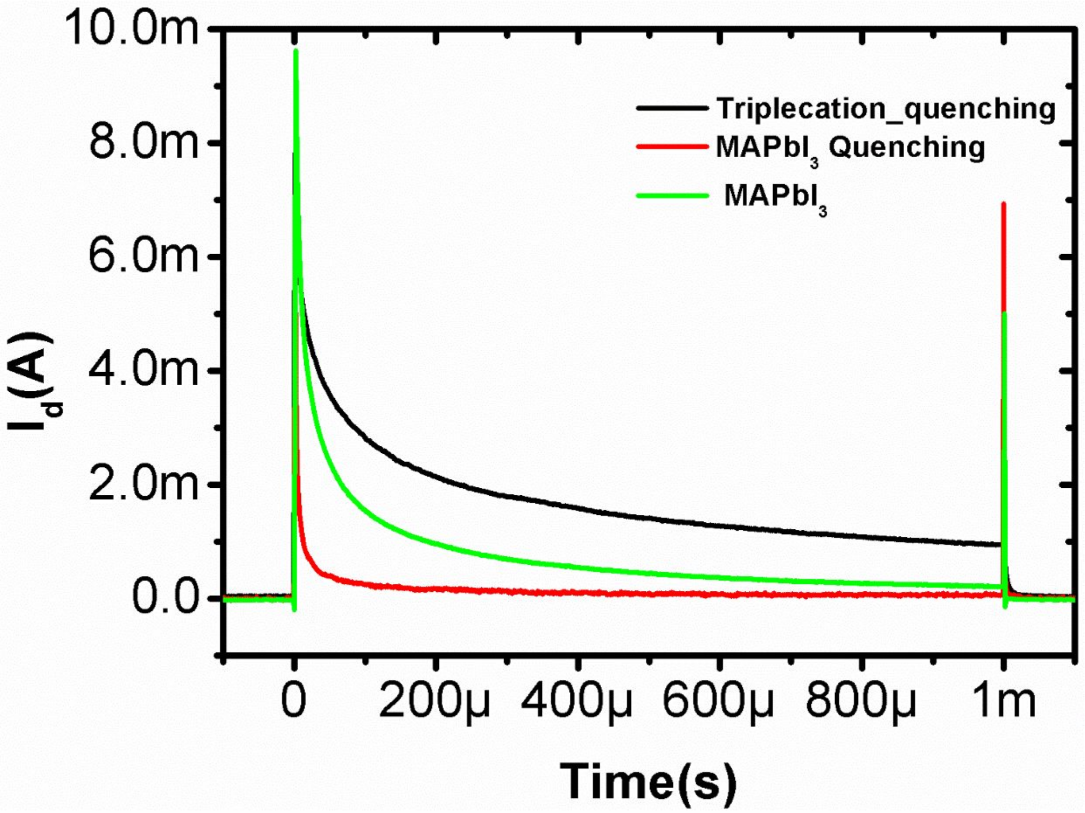

Figure S5. Transient measurements comparison of different composition and processing methods: black-triple cation, red- $MAPbI_3$ quenching, green- $MAPbI_3$ reference

Fig S5 clearly shows the decay of drain current (Id) with increment of pulse width, regardless of different composition and processing methods. This suggests that decay of the drain current with pulse width is consistent in all transient measurements. These transient measurements can also further aid in more understanding of charge transport in perovskite.

Section S2.7. Reliability tests by different modes

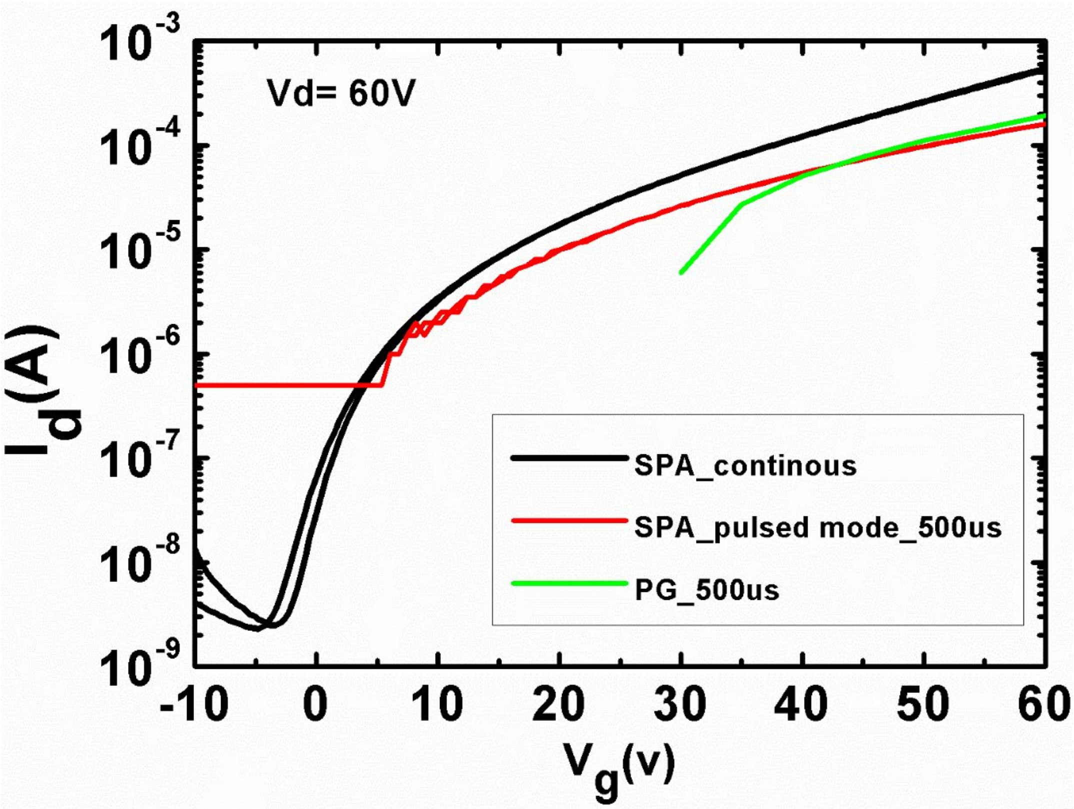

Figure S6. N2200 transfer characteristics measured from black-SPA continuous mode, red-SPA pulsed mode(500us), green-PG(500us)

Above transfer characteristics of organic transistors were done to show the reliable/genuine measurements done in this report. Measurement were referred here with organic transistors due to

their standard characteristics with transient measurements. Current by all measurements follow the similar trend however they are slight variation of currents levels may be due to self-heating effects during different operation mode. Fig S6 also shows the technical limitations of the off-current of pulsed mode in comparison to the continuous mode.

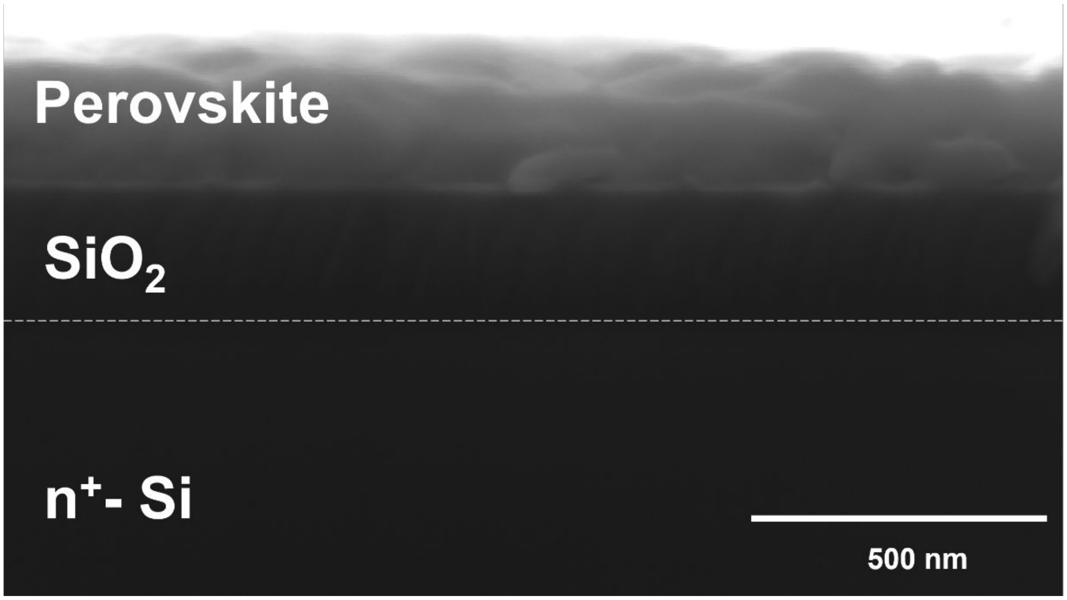

Figure S7: Cross-sectional Images on Fraunhofer substrate, dashed white line is place between SiO2 and Si for the better understanding of interace

Section S2.8. Comparison with Different Device Geometry and Hysteresis Characterization

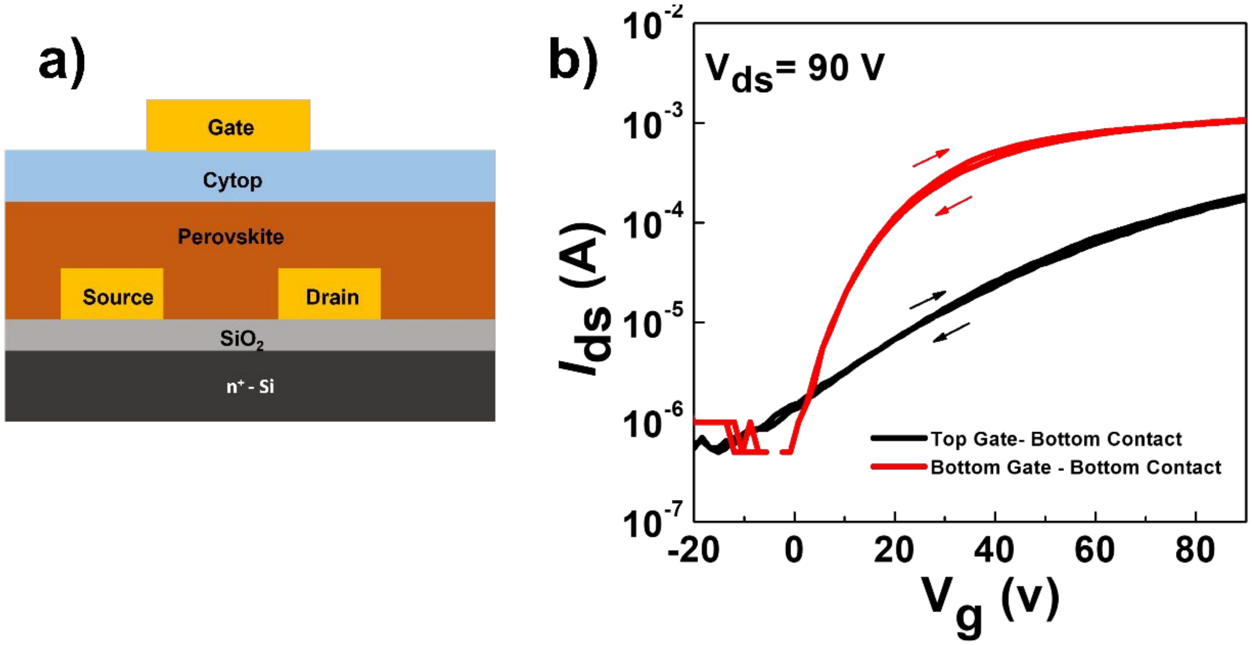

Figure S8. a) Device schematic of bottom gate bottom contact (BGBC) geometry and top gate bottom contact (TGBC) geometry. b) Typical transfer characteristics obtained respectively with the two different architectures, measured utilizing pulsed mode operation with pulses width of 500 μs.

We have performed comparative pulsed mode transfer measurements on bottom gate bottom contact (BGBC) geometry and top gate bottom contact (TGBC) geometry. The latter is realized on the same substrates and source-drain contacts, but with a top CYTOP dielectric with a gold

gate. It is evident that BGBC geometry achieves higher currents in comparison to the TGBC geometry. This could be attributed to more controlled and uniform growth of perovskite at SiO_2 gate and semiconductor interface. Also, the low hysteresis achieved in both cases can be appreciated.

Section S2.9. Gate Leakage Current

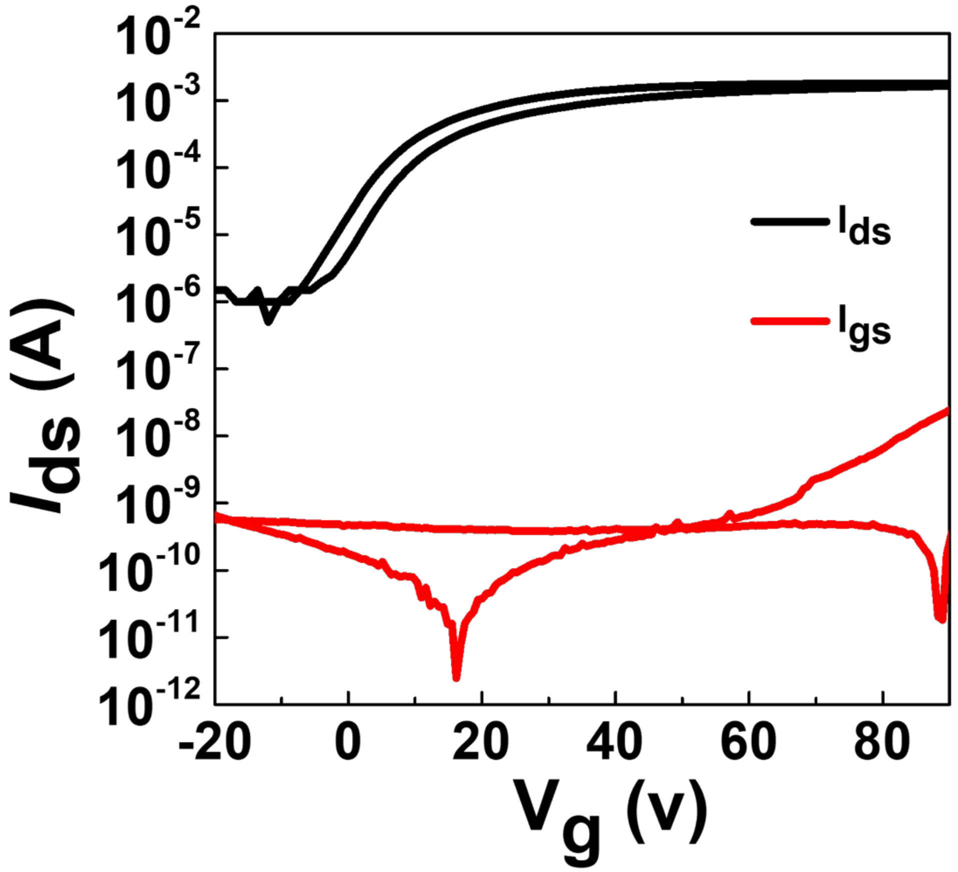

Figure S9. Transfer curve of the devices with drain current (I_{ds}), Gate leakage current (I_{gs}) representing black and red color curves respectively.

References:

1. M. Kim, S. G. Motti, R. Sorrentino and A. Petrozza, *Energy & Environmental Science*, 2018, **11**, 2609-2619.
2. A. R. b. M. Yusoff, H. P. Kim, X. Li, J. Kim, J. Jang and M. K. Nazeeruddin, *Advanced Materials*, 2017, **29**, 1602940.
3. H. H. Choi, K. Cho, C. D. Frisbie, H. Sirringhaus and V. Podzorov, *Nature Materials*, 2018, **17**, 2-7.
